# The longitudinal relationship between circulating concentrations of C-reactive protein, interleukin-6 and interleukin-10 in patients undergoing resection for renal cancer

**DOI:** 10.1038/sj.bjc.6603387

**Published:** 2006-09-26

**Authors:** S Ramsey, G W A Lamb, M Aitchison, D C McMillan

**Affiliations:** 1 Department of Urology, Gartnavel General Hospital, 1053 Great Western Rd, Glasgow G12 OYN, UK; 2 University Department of Surgery, Royal Infirmary, Glasgow G31 ER, UK

**Keywords:** renal cancer, nephrectomy, C-reactive protein, interleukin-6, interleukin-10, cancer-specific survival

## Abstract

The systemic inflammatory response, as evidenced by elevated circulating concentrations of C-reactive protein, is a stage-independent prognostic factor in patients undergoing curative nephrectomy for localised renal cancer. However, it is not clear whether the systemic inflammatory response arises from the tumour *per se* or as a result of an impaired immune cytokine response. The aim of the present study was to examine C-reactive protein, interleukin-6 and interleukin-10 concentrations before and following curative resection of renal cancer. Sixty-four patients with malignant renal disease and 12 with benign disease, undergoing resection were studied. Preoperatively, a blood sample was collected for routine laboratory analysis with a further sample stored before analysis of interleukin-6 and interleukin-10 using an enzyme-linked immunosorbent assay (ELISA) technique. The blood sampling procedure and analyses were repeated at approximately 3 months following resection. Circulating concentrations of both interleukin-6 and interleukin (*P*⩽0.01) were higher and a greater proportion were elevated (*P*<0.05) in malignant compared with benign disease. The renal cancer patients were grouped according to whether they had evidence of a systemic inflammatory response. In the inflammatory group T stage was higher (*P*<0.01), both interleukin-6 and interleukin-10 concentrations were higher (*P*<0.001) and elevated (*P*<0.10) compared with the non-inflammatory group. Tumour volume was weakly correlated with C-reactive protein (*r*^2^=0.20, *P*=0.002), interleukin-6 (*r*^2^=0.20, *P*=0.002) and interleukin-10 (*r*^2^=0.24, *P*=0.001). Following nephrectomy the proportion of patients with elevated C-reactive protein, interleukin-6 and interleukin-10 concentrations did not alter significantly. An elevated preoperative C-reactive protein was associated with increased tumour stage, interleukin-6 and interleukin-10 concentrations. However, resection of the primary tumour did not appear to be associated with significant normalisation of circulating concentrations of C-reactive protein, interleukin-6 or interleukin-10. Therefore, the presence of systemic inflammatory response is unlikely to be solely be determined by the tumour itself, but may be as a result of an impaired immune cytokine response in patients with renal cancer.

Renal cell cancer, although the 12th most common cause of cancer death is one of the most lethal urological cancers. Each year in the UK, there are approximately 3500 new cases and approximately 30% of patients present with metastases. Overall survival is poor; even in those who undergo potentially curative resection, only approximately half survive 5 years (Cancerstats, www.cancerresearchuk.org).

It has recently become clear that the systemic inflammatory response, as evidenced by elevated circulating concentrations of C-reactive protein, is an important prognostic factor independent of tumour stage in patients undergoing potentially curative surgery for a number of solid tumours including colorectal (Nielsen *et al*, 2000; McMillan *et al*, 2003); gastro-oesophageal (Ikeda *et al*, 2003; Crumley *et al*, 2006), pancreatic (Jamieson *et al*, 2005) and urinary bladder (Hilmy *et al*, 2005) cancers.

It has also been shown that an elevated circulating C-reactive protein concentration is associated with a poor prognosis in patients with metastatic renal cancer (Atzpodien *et al*, 2003; Bromwich *et al*, 2004; Casamassima *et al*, 2005). Furthermore, an elevated C-reactive protein concentration has also been shown to be associated with poor survival, independent of stage and grade, in patients undergoing potentially curative resection for renal cancer (Masuda *et al*, 1998; Lamb *et al*, 2006).

However, the basis of the independent relationship between an elevated C-reactive protein concentration and poor survival in renal cancer is not clear. Specifically, it is not clear whether the systemic inflammatory response arises from the tumour *per se* or as a result of an impaired immune cytokine response. Interleukin-6 and interleukin-10 are likely to be key cytokines in such a response as they appear to have stimulant and suppressive actions, respectively, on immune cells, in particular T-lymphocytes (Gabay and Kushner, 1999; Jee *et al*, 2001; Trikha *et al*, 2003). Interleukin-6 is recognised as an autocrine growth factor for tumours, but also has a tumour suppressive role in promoting anti-tumour activity of macrophages. (Trikha *et al*, 2003). More recently, interleukin-10 has been recognised to be an important immunosuppressive cytokine for the Th1 anti-tumour response and may be important in determining tumour growth and metastases (Mocellin *et al*, 2005).

The aim of the present study was to examine C-reactive protein, interleukin-6 and interleukin-10 concentrations before and following curative resection of renal cancer.

## PATIENTS AND METHODS

Patients with benign and malignant renal disease, who underwent resection between March 2003 and October 2005 in the North Glasgow NHS Trust were included in the study. Patients were staged pathologically according to the 1997 UICC TNM classification of renal tumours (Sobin and Wittekind, 1997). Tumours were graded according to criteria set out by Fuhrman *et al* (1982). Clinical stage and performance status (Eastern Cooperative Oncology Group, ECOG-ps) were recorded before surgery.

The Research Ethics Committee of North Glasgow NHS Trust approved the study.

### Experimental design

A blood sample was collected before resection for routine laboratory analysis of haemoglobin, white cell count, percentage lymphocyte count, albumin and C-reactive protein. The limit of detection of the assay was a C-reactive protein concentration lower than 6 mg l^−1^. The inter- and intra-assay variability of haemoglobin, white cell count, albumin and C-reactive, protein were less than 10%. A C-reactive protein concentration of greater than 10 mg l^−1^ was considered to indicate the presence of systemic inflammatory response (O'Gorman *et al*, 2000; McMillan *et al*, 2001). A further blood sample taken before surgery was centrifuged and the serum stored at −80°C before analysis of interleukin-6 and interleukin-10. Circulating concentrations of these cytokines were measured using an enzyme-linked immunosorbent assay (ELISA) technique. The minimum detectable concentrations were 2 pg ml^−1^ for interleukin-6 and 4 pg ml^−1^ for interleukin-10 (Quantikine ELISA, R&D Systems Europe Ltd, Abingdon, UK). Inter- and intra-assay variability were less than 10% for both assays.

A second blood sample was obtained approximately 3 months following nephrectomy for routine laboratory analysis and cytokine quantification using the methods above.

### Statistics

Data are presented as median and range. Comparisons between groups of patients were carried out using contingency table analysis (*χ*^2^) as appropriate. Cytokine concentrations below the threshold of sensitivity of the respective assays were expressed as equal to this threshold. Where appropriate, data were tested for statistical significance using Mann–Whitney *U* test and the Wilcoxon signed rank test. As the distribution of C-reactive protein and the cytokines were skewed, they were logarithmically transformed before stepwise multiple regression analysis for the examination of independent associations with C-reactive protein. Univariate survival analysis was performed using the Kaplan–Meier method with the log-rank test. Deaths up to the end of March 2006 were included in the analysis. Analysis was performed using SPSS software (SPSS Inc., Chicago, IL, USA).

## RESULTS

The clinicopathological characteristics of patients who underwent resection for benign (*n*=12) and malignant (*n*=64) renal disease are shown in Table 1. Age, sex, ECOG-ps, haemoglobin, white cell count, percentage lymphocyte count, albumin and C-reactive protein were similar in the two groups. Circulating concentrations of both interleukin-6 and interleukin-10 (*P*⩽0.01) were higher and a greater proportion were elevated (*P*<0.05) in malignant compared with benign disease.

The renal cancer patients were grouped according to whether they had evidence of a systemic inflammatory response before nephrectomy (C-reactive protein >10 mg l^−1^, Table 2). The groups were similar in terms of age, sex, tumour volume, Fuhrman grade, white cell count and percentage lymphocytes. In the inflammatory group T stage was higher (*P*<0.01), number of cytoreductive operations greater (*P*<0.05), both interleukin-6 and interleukin-10 concentrations were higher (*P*<0.001) and elevated (*P*<0.10) compared with the non-inflammatory group. In contrast, haemoglobin (*P*<0.01) and albumin (*P*<0.10) concentrations and ECOG-ps (*P*<0.05) were lower in the inflammatory group. Tumour volume was correlated with C-reactive protein (*r*^2^=0.20, *P*=0.002), interleukin-6 (*r*^2^=0.20, *P*=0.002) and interleukin-10 (*r*^2^=0.24, *P*=0.001).

The minimum follow-up was 7 months or until date of death; the median follow-up of the survivors was 25 months. During this period, 15 (20%) patients died: 11 patients of their cancer and four of intercurrent disease. On univariate analysis, an elevated C-reactive protein concentration before resection was associated with reduction in cancer-specific survival (*P*=0.014).

**Figure 1:**
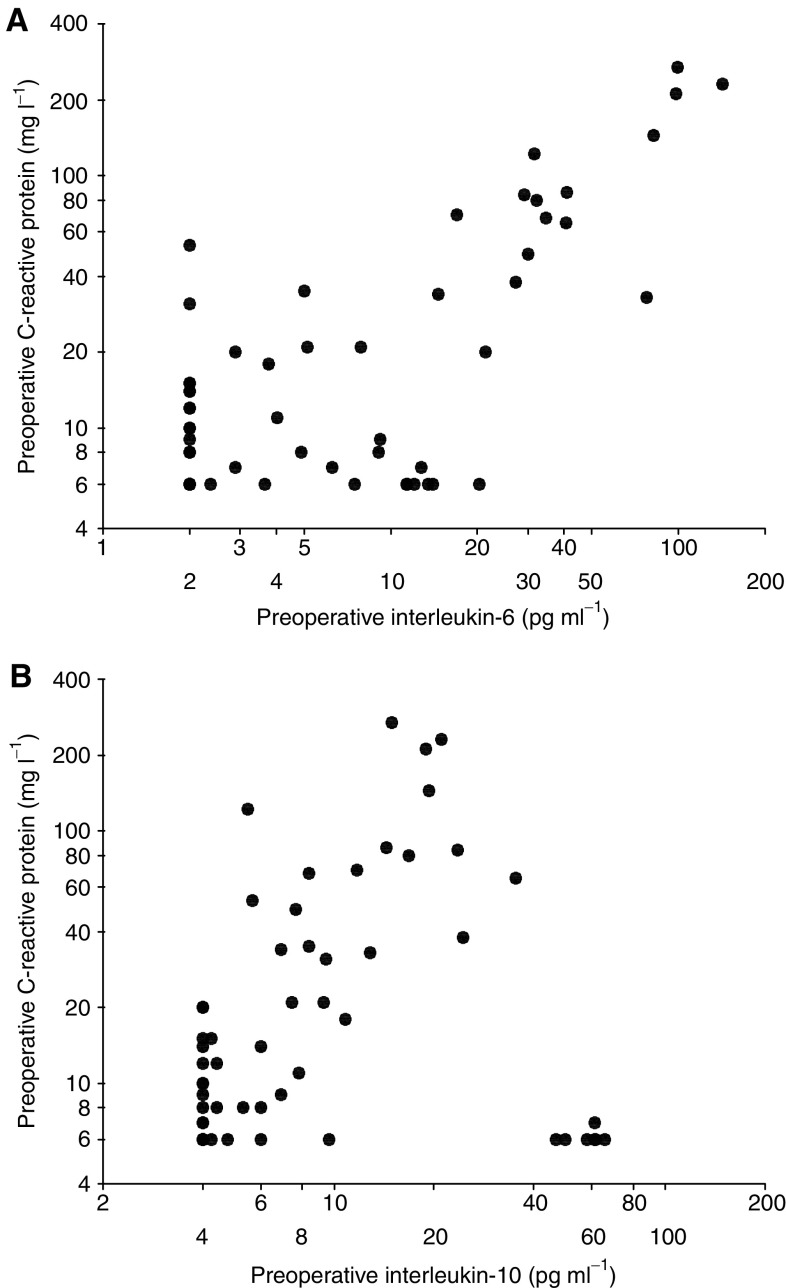
(**A**) Relationship between circulating concentrations of interleukin-6 and C-reactive protein in patients with renal cancer. (**B**) Relationship between circulating concentrations of interleukin-10 and C-reactive protein in patients with renal cancer.

In the cancer patients who had detectable circulating preoperative C-reactive protein concentrations (*n*=41), log-transformed concentrations of C-reactive protein were significantly correlated with interleukin-6 (*r*^2^=0.62, *P*<0.001, Figure 1A) and interleukin-10 (*r*^2^=0.33, *P*<0.001, Figure 1B). On multiple regression analysis of both interleukin-6 and interleukin-10 on C-reactive protein, only interleukin-6 (*r*^2^=0.63, *P*<0.001) retained independent significance. Interleukin-6 was significantly correlated with IL-10 (*r*^2^=0.49, *P*<0.001).

The clinicopathological characteristics of the patients who had undergone a potentially curative operation, before and approximately 3 months following nephrectomy, are shown in Table 3. The proportion of patients with a low percentage lymphocyte count, albumin and an elevated C-reactive protein concentration did not change significantly over this period. In contrast, there was a fall in performance status (*P*<0.01), haemoglobin (*P*<0.01) and an increase in white cell count during this period. Changes in interleukin-6 and interleukin-10 concentrations did not reach statistical significance.

In the cancer patients who had detectable circulating postoperative C-reactive protein concentrations (*n*=35), log-transformed concentrations of C-reactive protein were significantly correlated with those of interleukin-6 (*r*^2^=0.66, *P*<0.001) and interleukin-10 (*r*^2^=0.33, *P*<0.001). On multiple regression analysis of both interleukin-6 and interleukin-10 on C-reactive protein, only interleukin-6 (*r*^2^=0.66, *P*<0.001) retained independent significance. Interleukin-6 was significantly correlated with interleukin-10 (*r*^2^=0.51, *P*<0.001).

## DISCUSSION

In the present study, both interleukin-6 and interleukin-10 concentrations were greater in malignant compared with benign renal disease. Furthermore, both were directly associated with C-reactive protein and did not appear to normalise on resection of the primary renal tumour.

These results appear to contradict the report of Ljungberg *et al* (1995) who, in a similar study design of 56 patients with stage I renal cancer reported that a number of acute phase proteins including C-reactive protein fell significantly approximately 6 months after resection. However, in the present study, when the analysis was confined to those patients with stage I disease, neither C-reactive protein, interleukin-6 or interleukin-10 appeared to normalise on resection of the primary tumour.

Galizia *et al* (2002) in a similar study design in 50 patients with colon reported that that both interleukin-6 and interleukin-10 concentrations fell by day 16 following resection. However, it was of interest that, in their study, the median concentrations of interleukin-6 and interleukin-10, before surgery, were higher (8 and 15 pg ml^−1^, respectively) compared with the results (3 and 5 pg ml^−1^, respectively) in the present study. Nevertheless, consistent with the present study Galizia *et al* (2002) observed that the majority of patients did not normalise their cytokine concentrations following radical resection.

The basis of the discrepancies between the present and previous studies is not clear. Nevertheless, the results of the present study are consistent with previous pre-/postoperative C-reactive protein findings in colorectal, pancreatic and bladder cancer (McMillan *et al*, 2003; Jamieson *et al*, 2005; Hilmy *et al*, 2005). Furthermore, if there were to be a significant conversion rate from a systemic inflammatory state (C-reactive protein >10 mg l^−1^) to a non-inflammatory state (C-reactive protein ⩽10 mg l^−1^) following resection then the prognostic value of markers of the systemic inflammatory response would be significantly degraded.

The elevated circulating concentrations of interleukin-6 and interleukin-10 following resection of renal cancer may reflect a continuing Th2 cytokine response as increased intra-tumoural CD4+ T-lymphocyte infiltrate has been shown to be associated with poor outcome, independent of grade, in patients with renal clear-cell cancer (Bromwich *et al*, 2003). This would be consistent with the observations in the present study that circulating interleukin-6 and interleukin-10 concentrations were not strongly correlated with tumour volume but were similarly correlated with each other before and after resection of the renal tumour. Moderation of this cytokine response may be important in the regulation of the systemic inflammatory response and warrants further clinical investigation.

Given the considerable variability of the effect of resection on C-reactive protein, interleukin-6 and interleukin-10 seen in the present study it would require a much larger study to absolutely preclude the possibility that surgical resection of renal cancer does not reduce C-reactive protein, interleukin-6 and interleukin-10 concentrations. Nevertheless, the results of the present study would suggest that the presence of systemic inflammatory response is not solely determined by the elaboration of cytokines from the tumour.

In summary, an elevated preoperative C-reactive protein was associated with increased tumour stage, interleukin-6 and interleukin-10 concentrations. However, resection of the primary tumour did not appear to be associated with significant normalisation of circulating concentrations of C-reactive protein, interleukin-6 or interleukin-10. Therefore, the presence of systemic inflammatory response is unlikely to be solely be determined by the tumour itself, but may be as a result of an impaired immune cytokine response in patients with renal cancer.

## Figures and Tables

**Table 1:** Clinicopathological characteristics in patients with benign and malignant renal disease before nephrectomy

	**Benign disease (n=12)**	**Renal cancer (n=64)**	***P*-value**
Age group (⩽60/>60 years)	9/3	35/29	0.194
Sex (male/female)	4/8	42/22	0.037
T stage (1/2/3/4)		27/4/21/12	
Tumour volume (cm^3^)		112 (1–4864)	
Fuhrman grade (1/2/3/4)		8/16/21/13	
Operation (curative/cytoreductive)		54/10	
ECOG-PS (0/1)	12/0	53/11	0.123
Haemoglobin (⩾12/<12 g dl^−1^)	9/3	50/14	0.813
White cell count			
(<8.5/8.5–11.0/>11.0 10^9^ l^−1^)	7/5/0	48/13/3	0.486
Lymphocyte percentage			
(20–40/12.0–19.9/0–11.9%)	10/2/0	43/15/6	0.199
Albumin (⩾35/<35 g l^−1^)	11/0	59/3	0.459
C-reactive protein (⩽10/>10 mg l^−1^)	8/4	34/30	0.390
Interleukin-6 (pg ml^−1^)	<2 (<2–19)	4 (<2–142)	0.007
Interleukin-6 (⩽4/>4 pg ml^−1^)	10/1	33/31	0.015
Interleukin-10 (pg ml^−1^)	<4 (<4–7)	6 (<4–66)	0.012
Interleukin-10 (⩽10/>10 pg ml^−1^)	12/0	45/19	0.030

ECOG, Eastern Cooperative Oncology Group.

Median (range).

**Table 2:** Clinicopathological characteristics of inflammatory and non-inflammatory patients with renal cancer before nephrectomy

	**C-reactive protein ⩽10 mg l^−1^ (n=34) median <6 mg l^−1^**	**C-reactive protein>10 mg l^−1^ (n=30) median 35 mg l^−1^**	***P*-value**
Age group (⩽60/>60 years)	17/17	18/12	0.426
Sex (male/female)	23/11	19/11	0.719
T stage (1/2/3/4)	19/2/11/2	8/2/10/10	0.005
Tumour volume (cm^3^)	64 (1–1331)	179 (9–4864)	0.123
Fuhrman grade (1/2/3/4)	5/11/9/6	3/5/12/7	0.193
Operation (curative/cytoreductive)	32/2	22/8	0.023
ECOG-PS (0/1)	32/2	21/9	0.011
Haemoglobin (⩾12/<12 g dl^−1^)	31/3	19/11	0.008
White cell count			
(<8.5/8.5–11.0/>11.0 10^9^ l^−1^)	26/8/0	22/5/3	0.344
Lymphocyte percentage			
(20–40/12.0–19.9/0–11.9%)	26/6/2	17/9/4	0.100
Albumin (⩾35/<35 g l^−1^)	33/0	26/3	0.060
Interleukin-6 (pg ml^−1^)	<2 (<2–20)	16 (<2–142)	0.001
Interleukin-6 (⩽4/>4 pg ml^−1^)	22/12	11/19	0.026
Interleukin-10 (pg ml^−1^)	<4 (<4–66)	8 (<4–35)	0.013
Interleukin-10 (⩽10/>10 pg ml^−1^)	27/7	18/12	0.092
Cancer–specific survival (months)^a^	34.8 (32.6–37.1)	28.1 (23.1–33.0)	0.014

ECOG, Eastern Cooperative Oncology Group.

Median (range).

a Mean (95%CI).

**Table 3:** Clinicopathological characteristics of patients with renal cancer before and approximately 3 months following potentially curative nephrectomy for localised renal cancer

	**Pre-nephrectomy (n=54)**	**Post-nephrectomy (n=54)**	***P*-value**
Age group (⩽60/>60 years)	26/28		
Sex (male/female)	34/20		
T stage (1/2/3/4)	27/4/21/2		
Tumour volume (cm^3^)	112 (1–4864)		
Fuhrman grade (1/2/3/4)	8/14/16/10		
			
ECOG-PS (0/1)	48/6	39/15	0.007
Haemoglobin (⩾12/<12 g dl^−1^)	45/9	33/21	0.007
White cell count			
(<8.5/8.5–11.0/>11.0 10^9^ l^−1^)	41/11/2	31/20/3	0.029
Lymphocyte percentage			
(20–40/12.0–19.9/0–11.9%)	39/11/4	37/12/4	0.874
Albumin (⩾35/<35 g l^−1^)^a^	51/2	45/1	0.317
C-reactive protein (<10/>10 mg l^−1^)	32/22	35/19	0.439
Interleukin-6 (pg ml^−1^)^a^	3 (<2–99)	<2 (<2–44)	0.227
	10 (18)^b^	6 (9)^b^	
Interleukin-6 (⩽4/>4 pg ml^−1^)^a^	31/23	28/9	0.059
Interleukin-10 (pg ml^−1^)^a^	5 (<4–66)	6 (<4–112)	0.056
	12 (17)^b^	18 (26)^b^	
Interleukin-10 (⩽10/>10 pg ml^−1^)^a^	42/12	24/13	0.257

ECOG, Eastern Cooperative Oncology Group.

a Post-nephrectomy *n*=37.

b Mean (s.d.), median (range).
